# A meta-analysis of the secondary fractures for osteoporotic vertebral compression fractures after percutaneous vertebroplasty

**DOI:** 10.1097/MD.0000000000025396

**Published:** 2021-04-23

**Authors:** Gongwei Zhai, Ang Li, Binfeng Liu, Dongbo Lv, Jingyi Zhang, Weichao Sheng, Guang Yang, YanZheng Gao

**Affiliations:** a People's Hospital of Henan University of Chinese Medicine, People's Hospital of Zhengzhou; b Department of Surgery of Spine and Spinal Cord, Henan Provincial People's Hospital, Henan Province Intelligent Orthopedic Technology Innovation and Transformation International Joint Laboratory, Henan Key Laboratory for Intelligent Precision Orthopedics, People's Hospital of Zhengzhou University, People's Hospital of Henan University, Henan Zhengzhou; c Zhengzhou University People's Hospital, Henan Provincial People's Hospital, China.

**Keywords:** osteoporosis, secondary fracture, spinal fractures, systematic review, vertebroplasty

## Abstract

To identify the risk factors of the secondary fractures for osteoporotic vertebral compression fractures (OVCFs) after percutaneous vertebroplasty (PVP).

We conducted a search of relevant articles using Cochrane Library, PubMed, Medline, Science Direct, Embase, the Web of Science and other databases. The time range we retrieved from establishment of the electronic database to November 2017. Gray studies were found in the references of included literature reports. STATA version 11.0 (Stata Corporation, College Station, Texas) was used to analyze the pooled data.

Fourteen studies involving 1910 patients, 395 of whom had fracture secondary to the surgery were included in this meta-analysis. The result of meta-analyses showed the risk factors of the secondary fractures for OVCFs after PVP was related to bone mineral density (BMD) [95%CI (−0.650, −0.164), SMD=−0.407, *P*=.001], cement leakage ((RR=0.596, 95%CI (0.444,0.798), *P* = .001)), and kyphosis after primary operation ((SMD=0.741, 95%CI (0.449,1.032), *P* = .000)), but not to gender, age, body mass index, cement volume, thoracolumbar spine, and cement injection approaches.

Bone mineral density, cement leakage, and kyphosis after primary operation are the risk factors closely correlative to the secondary fracture after PVP. There have not been enough evidences to support the association between the secondary fracture and gender, age, body mass index, cement volume, thoracolumbar spine, and cement injection approaches.

## 1 Introduction

Osteoporosis is a systemic disease with progressive dicalcium and bone structure abnormality, which could result in compression fracture even under a slight external force (falling, lifting heavy objects, coughing violently).^[1,2]^ Osteoporotic fracture often occurred in spine, hip, and distal radius. In addition, osteoporotic vertebral compression fracture (OVCF) is the most common fracture, which accounted for more than 1/3.^[3–5]^ Patients with OVCF have a series of symptoms such as back pain and kyphosis, which can seriously affect their life quality.^[6]^ Furthermore, the type of fracture has a slow healing, accompanied by a high rate of disability and death.^[7]^ The traditional treatment methods include bed rest, drug analgesia, or bracing external fixation, which could resulted in a vicious circle of decalcification of bone, progresses of severe pain, kyphotic deformity, and increasing mortality.^[8]^ For the treatment of OVCF, percutaneous vertebrolplasty (PVP) as an effect technology has minimally invasive pain and rapid recovery. Through injecting bone cement into the target vertebral body, the vertebroplasty was successful performed. Accumulating physicians and patients have been obtained the identification of surgical procedure of PVP.^[9,10]^ However, numerous clinical data have been confirmed that the incidence of nonsurgical vertebral fractures was 8% to 52%, and 41% to 69% of the secondary fractures occurred in the adjacent segment of the vertebral body.^[11–13]^ Lin et al^[14]^ believed that the stiffness and other biomechanical factors of the injured vertebral bone cement injection lead to changes in biomechanics of the whole spine, resulting in significant changes in the pressure of adjacent vertebral body. Though, the bulk of the data showed that the gender (significantly more women than men), age (mainly 60–80 years old), bone mineral density (BMD), cement volume, cement leakage are the underlying inducements. PVP-related risk factors of postoperative secondary fractures are not consistent in OVCF patients.^[15,16]^ Therefore, we collected literatures on the related factors of vertebral fracture after PVP of OVCF patients, also assessed the effects of these factors on vertebral secondary fractures through meta-analysis.

## 2 Materials and methods

### 2.1 Literature and search strategy

The retrieved object is the research literature on the analysis of secondary fractures of OVCF following PVP operation published publicly in the electronic databases including PubMed, Cochrane Library, EMBASE, and Web of Science from 1966 to November 2017. The search strategy was based on combination with Boolean logic: osteoporosis, vertebral compression fracture, PVP, subsequent fracture, secondary fractures. In addition, the study of the appraisal reference list was manually checked to determine other potential eligibility tests. The process will iterate until more projects cannot be identified. The Meta-analysis is based on accepted PRISMA criteria (priority reporting items for systematic review and meta-analysis).

### 2.2 Inclusion and exclusion criteria

If the literature conforms to the following principles, these articles will be included in this meta-analysis:

1. OVCF patients undergoing PVP surgery;
2. analysis of related risk factors for postoperative patients with secondary fractures;
3. an official published RCTs or nonRCTs full-text English-written articles;
4. one or more adequate data of the outcomes could be conducted statistical analysis;

### 2.3 Data extraction and outcome measures

Two reviewers (Zhai Gongwei and Li Ang) extracted data from the included studies. The following essential information was captured: first author names, samples size, publication year, outcomes, study design, and other relevant data. The extracted data (range and size of the experiment, median, MD, and SD) are input into the designed standardized table. When there are differences of opinion, the controversies were settled by consensus or discussion with a 3rd author (Liu Binfeng). The outcome measurements were gender, age, body mass index (BMI), bone mineral density (BMD), cement volume, intradiscal cement, thoracolumbar spine, cement injection approach, kyphosis after primary operation.

### 2.4 Quality assessment and statistical analysis

Tool of Cochrane Bone, methodological index for non-randomized studies and the Joint and Muscle Trauma Group were used to evaluate the quality of RCT and nonRCTs. Literature quality assessment is conducted by 2 reviewers (BFL, AL). Consensus was reached through consultation for divergence. We used Stata version 11.0 (Stata Corporation, College Station, Texas) for statistical analysis. When *I*^*2*^ > 50%, we considered that the data had obvious heterogeneity, and meta-analysis was conducted using random effect model according to Cochrane Handbook for Systematic Reviews of Interventions (version 5.1.0). Otherwise, the fixed effect model is adopted. The continuous outcomes (age, BMI, BMD, cement volume, cement leakage) were expressed as 95% confidence interval (CIs) mean deviation (MD). For discontinuous various outcomes (gender, cement injection approach, kyphosis after primary operation, thoracolumbar spine) risk ratio (RR) with 95% CIs was used for evaluation.

### 2.5 Ethical statement

As all analyses were conducted with data from previously published studies, ethical approval was not necessary.

## 3 Results

### 3.1 Search results

**Figure 1:**
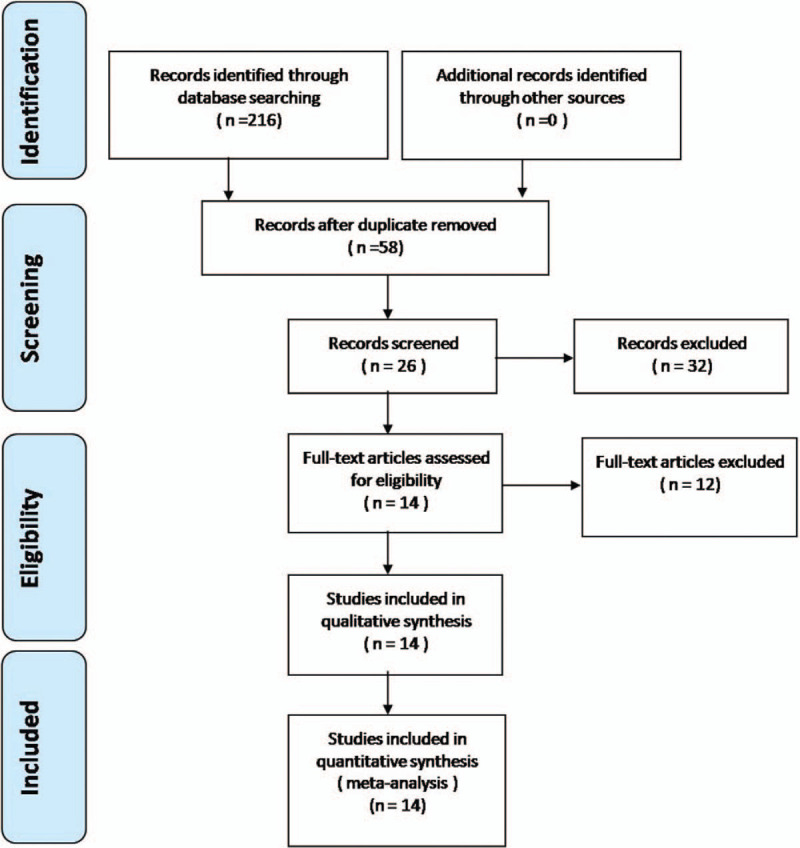
Flowchart of the study selection process.

A total of 58 studies were identified as potential literature reports. By scanning headings and abstracts, 44 reports were excluded according to eligibility criteria. No additional studies were obtained after review of the references. Finally, 14 nonrandomized controlled trials met the requirements of data extraction and meta-analysis. Table 1 Shows the characteristics of the trials. The article selection chart is shown in Figure 1.

**Table 1:** Cohort characteristics.

Studies	Year	Country	Fractures	Simple size	Age	Type	Follow-up	NOS
Lee WS	2006	Korea	38	244	Average 66.4	RCS	>48	7
Li YA	2012	China	63	166	Average 73.4	RCS	12–60	6
Lin WC	2008	China	14	29	56–77	RCS	—	8
Lo YP	2008	China	15	220	53–97	RCS	24–36	8
Lu K	2012	China	34	155	43–94	RCS	>24	8
Martinez	2013	Spain	17	57	73.6 ± 9.3	RCS	6–39	7
Ren HL	2015	China	21	182	49–91	RCS	6–60	8
Rho YJ	2012	Korea	27	147	49–93	RCS	12–73	8
Sun G	2014	China	37	175	70.3 ± 8.2	RCS	>12	8
Kang SK	2011	Korea	27	60	Average 70	RCS	>12	7
Lin H	2008	China	14	29	Average 73.8	RCS	22.4	8
Voormolen	2006	Dutch	16	66	46–88	RCS	>12	6
Ahn Y	2008	Korea	45	95	Average 69.3	RCS	—	8
Yoo CM	2012	Korea	49	244	48-93	RCS	0.2–61.7	8

### 3.2 Bone mineral density

**Figure 2:**
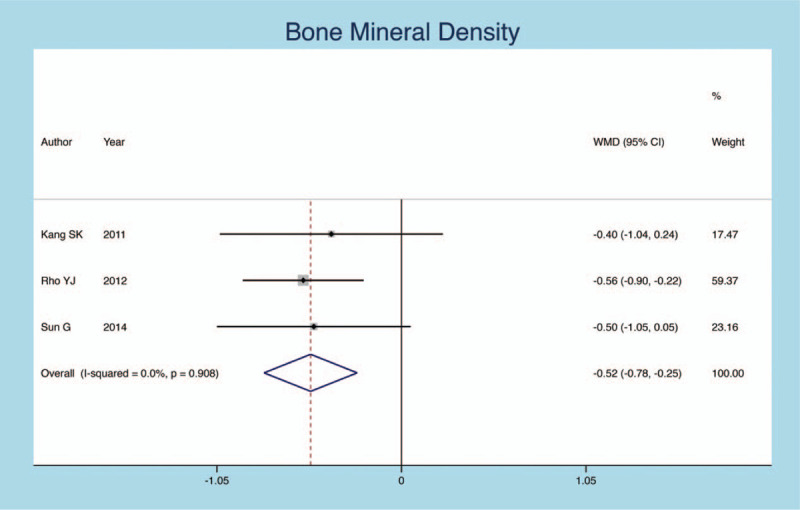
Forest plot diagram showing the BMD.

We extracted the BMD value from 3 included articles. The results show that BMD is a risk factor for secondary fracture after PVP (heterogeneity *P* = .564, *I*^*2*^ = 0.0%, SMD = −0.407, 95% CI: −0.650 to −0.164, *P* = .001; Fig. 2).

### 3.3 Cement leakage

**Figure 3:**
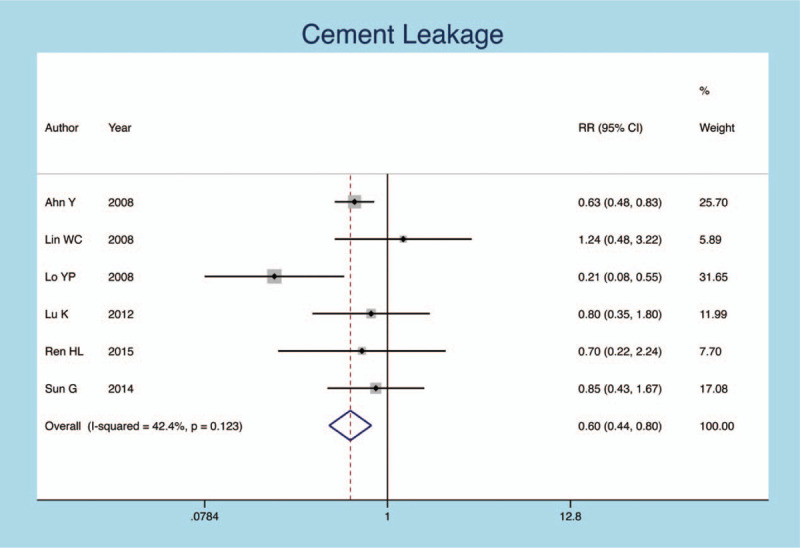
Forest plot diagram showing the cement leakage.

Six articles have been demonstrated the relationship between the cement leakage and secondary fractures rate. There is no significant heterogeneity in the statistical results of the pooled literature (*I*^*2*^ = 42.4%, *P* = .123). The result of the fixed effect model showed that the cement leakage could increase the incidence of new fractures (95% CI: 0.444–0. 798, RR = 0.596, *P* = .001; Fig. 3).

### 3.4 Kyphosis

**Figure 4:**
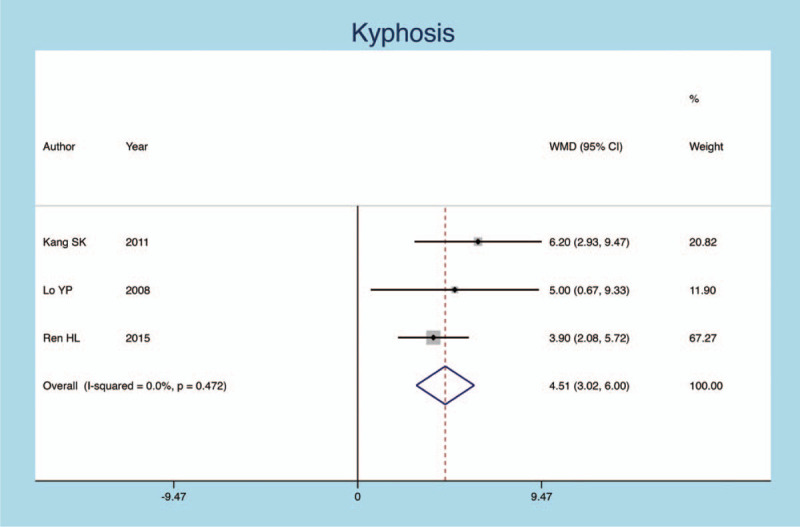
Forest plot diagram showing the kyphosis.

Three publications focus on the effect of postoperative kyphosis after primary operation on secondary fractures. Similar to the results described above, postoperative kyphosis angle of vertebra is closely related to the secondary fractures (heterogeneity *P* = .0000, *I*^*2*^ = 0.0%, SMD = 0.741, 95% CI: 0.449–1. 032, *P* = .0000; Fig. 4).

### 3.5 Gender

**Figure 5:**
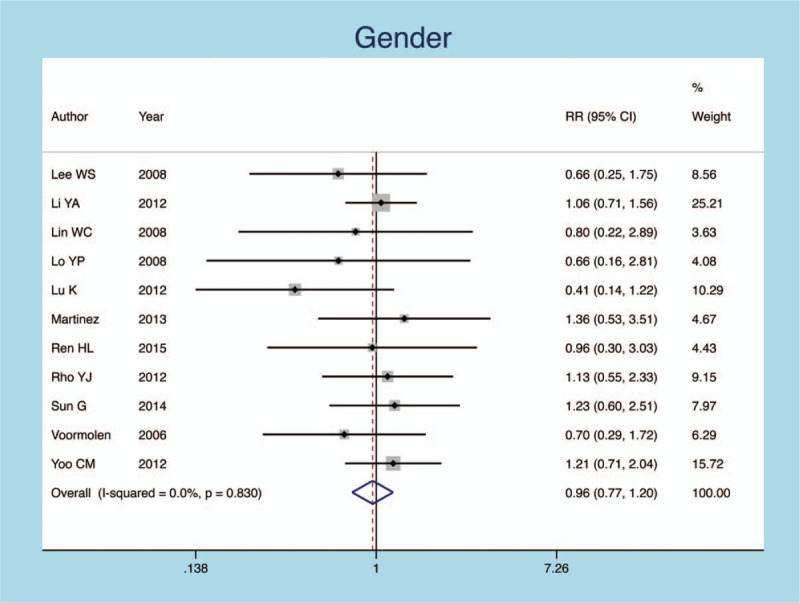
Forest plot diagram showing the gender.

In 5 publications, 93 female, and 248 males were included in our meta-analysis, respectively. There was no difference between the gender and the risk factor (heterogeneity *P* = .830, *I*^*2*^ = 0.0%, RR = 0.962, 95% CI: 0.768–1.204, *P* = .733; Fig. 5).

### 3.6 Age

**Figure 6:**
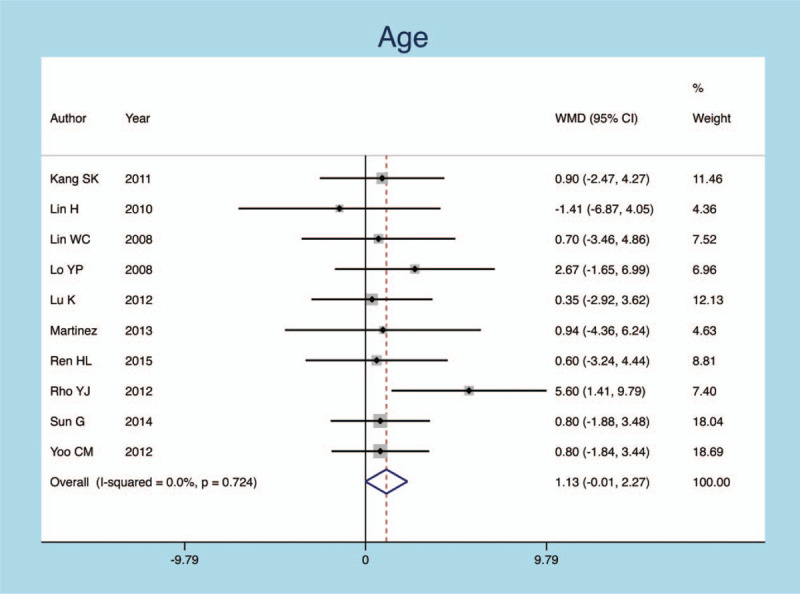
Forest plot diagram showing the age.

The meta-analysis result showed that age had no effect on postoperative new fractures of OVCF patients following PVP operation (heterogeneity *P* = .0000, *I*^*2*^ = 0.0%, SMD = 0.133, 95% CI: −0.006–0.271, *P* = .733; Fig. 6).

### 3.7 Cement volume

**Figure 7:**
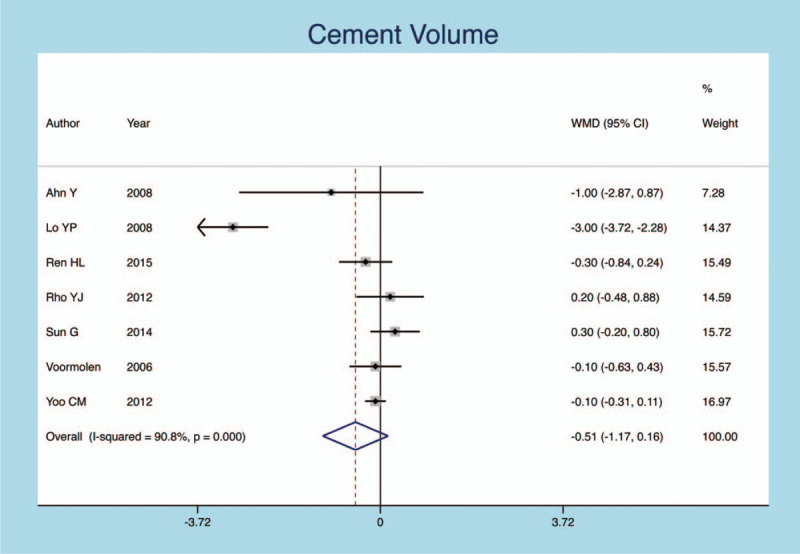
Forest plot diagram showing the cement volume.

No significant differences were observed between the cement volume and new fractures rate of OVCF patients following PVP operation (SMD = −0.245, 95% CI: −0.608–0.119; *P* = .187, Fig. 7) with obvious heterogeneity (heterogeneity *P* = .012, *I*^*2*^ =78.9%).

### 3.8 Body mass index

**Figure 8:**
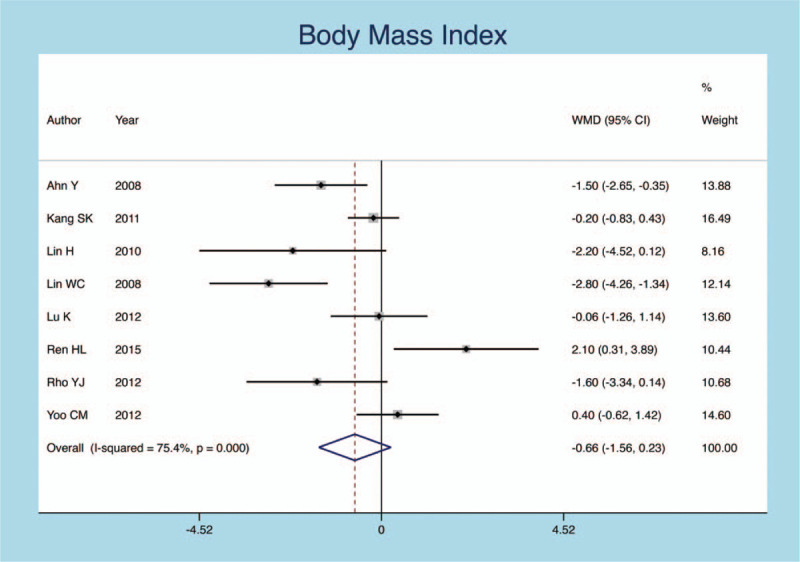
Forest plot diagram showing the BMI.

The BMI was reported in 8 inclusion studies. A random-effects model with obvious heterogeneity is established. (*I*^*2*^ = 74.4%, *P* = .1432). There was no significant statistical difference in BMI (SMD = −0.216, 95% CI: −0.526–0.094, *P* = .172; Fig. 8).

### 3.9 Thoracolumbar spine

**Figure 9:**
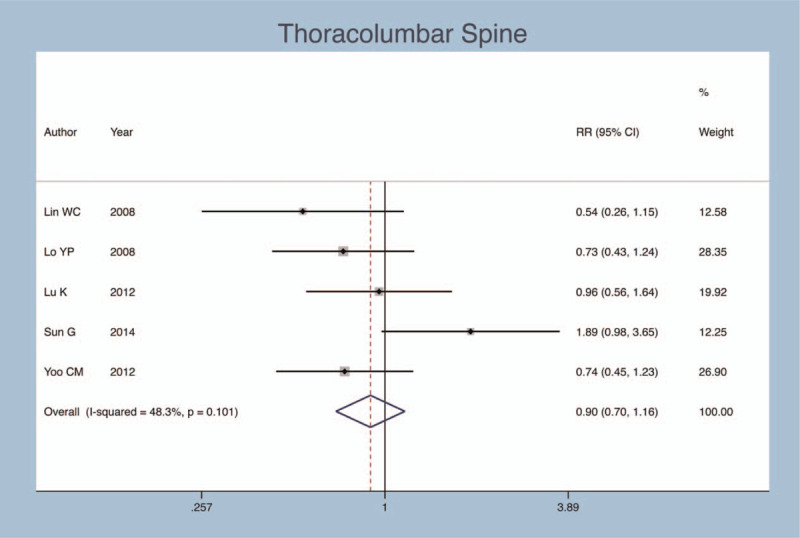
Forest plot diagram showing the thoracolumbar spine.

Five studies concentrate on whether the primary fracture in thoracic lumbar segment effects on postoperative secondary fractures of PVP operation. There was no obvious heterogeneity (*I*^*2*^ = 48.3%, *P* = .101); therefore, a fixed-effects model was adopted. Pooling the results demonstrated that primary fracture in thoracic lumbar segment has no effect on secondary fractures after PVP (RR = 0.898, 95% CI: 0.496–1.159, *P* = .409; Fig. 9).

### 3.10 Cement injection approach

**Figure 10:**
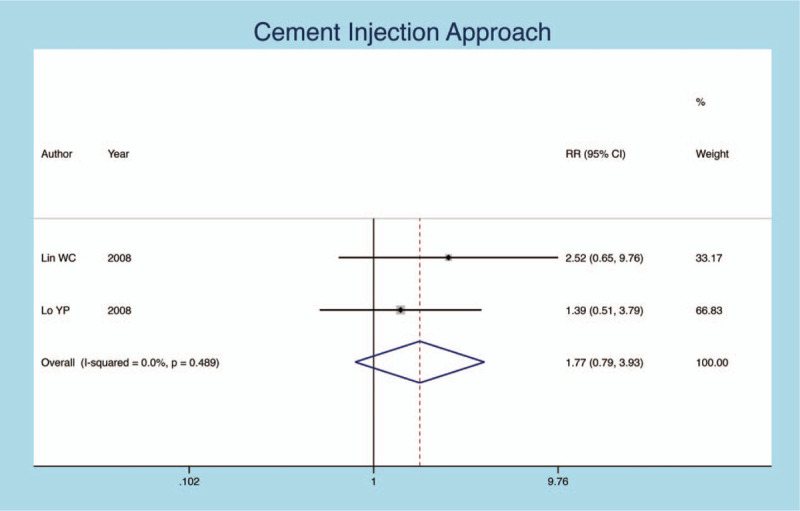
Forest plot diagram showing the cement injection approach.

Seven publications compare the secondary fracture rate of different cement injection approaches. The result show that different cement injection approaches are independent of secondary fractures (heterogeneity *P* = .000, *I*^*2*^ = 78.9%, RR = −0.245, 95% CI: −0.608–0.119, *P* = .0000; Fig. 10). All Meta-analysis results are illustrated in Table 2

**Table 2:** Meta-analysis results.

			Overall effect			Heterogeneity	
Outcome	Studies	Groups (s/N)	Effect estimate	95% CI	*P* value	I^2^ (%)	*P* value
BMD	3	91/291	−0.407	−0.650, −0.164	.001	0.0%	.564
Cement leakage	6	269/1070	0.596	0.444, 0. 798	.001	42.4%	.123
Kyphosis	3	28/8	0.741	0.449, 1. 032	.0000	0.0%	.0000
Gender	11	97/248	0.962	0.768,1.204	.733	0.0%	.830
Age	10	269/1070	0.133	−0.006, 0.271	.060	0.0%	.0000
Cement volume	7	181/919	−0.245	−0.608, 0.119	.187	78.9%	.1856
BMI	8	245/737	−0.216	−0.526, 0.094	.172	74.4%	.1432
Thoracolumbar spine	5	117/75	0.898	0.496,1.159	.409	48.3%	.101
Cement injection approach	7	63/499	−0.245	−0.608,0.119	.187	78.9%	.000

## 4 Discussion

The aging process of the population has accelerated osteoporosis as one of the common diseases that endanger the health of the elderly, and OVCF are the main complications of osteoporosis.^[5]^ According to the literature, OVCF accounts for about 45% of all osteoporotic fractures.^[17]^ PVP could not only rapidly and effectively relieve pain, but also shorten the hospitalization days and improve the life quality of OVCF patients.^[18]^ Though, PVP has been widely used in the treatment of OVCF, the problem of secondary fractures following PVP surgery is widely concerned. The incidence of new fractures following PVP reported in the study was 7.4% to 52%.^[19,20]^ However, PVP-related risk factors of postoperative secondary fractures are not consistent in OVCF patients. The results of our meta-analysis show that the new fractures after PVP for OVCF patients was related to BMD, cement leakage and kyphosis after primary operation, but not to gender, age, BMI, cement volume, thoracolumbar spine. or cement injection approaches.

Mudano et al^[21]^ believed that the risk of secondary fractures in patients after PVP is significantly higher than that of conservative treatment. By using spinal finite element model, other scholars^[22]^ observed that the bone cement injection could reduce the physiological concave of vertebral endplate. This process not only increased the vertebral body pressure by 19%, but also reduced the flexibility of local spinal joints, and the load of adjacent segments could increase by 17%. Another explanation for the secondary fractures is that increased daily activity in postoperative patients aggrandize the stress of the vertebral body, resulting in higher risk of secondary fractures.^[23]^

BMD as an important symbol of bone mass, which could reflect the degree of osteoporosis. One study^[23]^ showed that patients with lower BMD were more likely to have secondary fractures after PVP. One follow-up study of 104 cases of OVCF patients with PVP shows that there are 51.9% of patients of postoperative recurrence of adjacent vertebral fracture. The BMD of the fracture group was −3.52, the nonfracture group was −2.91. Logistic regression analysis showed that there was a negative correlation between BMD and the risk of fracture of adjacent vertebral bodies, suggesting that the lower BMD could cause higher risk of secondary fractures of the adjacent vertebral bodies.^[24]^ The research of Lu in 155 patients with PVP showed that the probability of new osteoporotic fractures within 2 years after the operation was 27.7%.^[25]^ Furthermore, BMD in the fracture group was significantly lower than that of no more fracture group (−3.07 vs −2.24, *P*<.05). The results of our meta-analysis confirmed that low BMD was a high risk factor for postoperative secondary fractures of OVCF patients. It also has a higher risk of secondary fractures even without PVP in OVCF patients. This risk may be the natural development of osteoporosis.

The association between the secondary fractures and the correction of vertebral kyphosis is uncertain. KANG et al^[16]^ found that 20 out of 27 cases of secondary fractures were surgical vertebral body fractures. The larger angle of the kyphosis preoperatively is a risk factor for fracture. At the same time, the correction of postoperative kyphosis led to the imbalance of the stress of the vertebral body, which also increases the risk of vertebral fracture. Another study reported by Lin^[14]^ considered that each degree rectification in the vertebral kyphosis could increase the risk of adjacent vertebral fractures by 9%. However, Lunt et al^[26]^ reported that kyphosis corrections could reduce the incidence of adjacent fractures. In addition, Robert et al^[27]^ believed that the severity of kyphosis was associated with a subsequent fracture of the adjacent vertebral body. The results of present meta-analysis shows that the incidence of secondary fractures in patients with larger postoperative vertebral kyphosis is higher. Under normal conditions, the compression load of the spine is perpendicular to the endplate of the vertebral body, and the progressive kyphosis will result in a change in the distribution of the spinal load, thus increasing the incidence of vertebral secondary fractures.^[23]^ Therefore, we believe it is more favorable to correct kyphotic deformity without causing complications.

During the operation of PVP, bone cement could be overflowed through fractures fissure of vertebral body when it is injected into bone cement.^[28,29]^ The study showed that the leakage of bone cement to the intervertebral space increased the risk of fracture of adjacent vertebral body.^[23,30]^ The following mechanism of bone cement leakage may lead to a recurrent fracture of the adjacent vertebral body:

1. when the bone cement leaks into the intervertebral space, the stress reduction of the injured vertebral disc leads to an increase in the stress of the adjacent vertebral body.
2. The leakage of bone cement could mechanically stimulate the endplate plate of the adjacent vertebral body, accelerate the degeneration of the disc, and further increase the risk of fracture of adjacent vertebral body. The results of our meta-analysis indicate that bone cement leakage could increase the incidence of vertebral secondary fractures.

The choice of unilateral or bilateral puncture in PVP operation is still controversial. Steinmann J et al^[31]^ hold the point that it has no statistically significant difference in the efficacy and mechanical analysis of PVP by unilateral or bilateral pedicle puncture. The study of VAN -MEIRHAEGHE^[32]^ found that the strength and stiffness of injured vertebrae could be restored by unilateral injection or bilateral injection, which had no significant effect on the stress of nonoperative vertebral body. The present meta-analysis demonstrate that cement leakage is not a risk factors for the new fractures to PVP for OVCF patients.

Kim DJ et al^[33]^ compared the different volume of bone cement injection in PVP operation showing that it has no correlation between the secondary fractures and volumes of bone cement injection. Our meta-analysis gets the same conclusion. In addition, some scholars believe that the vertebrae which close to the primary fracture site has higher risk, especially the vertebrae at the thoracolumbar junction.^[34,35]^ However, the result of meta-analysis shows that whether the primary fracture is located in the thoracolumbar segment has no effect on the secondary fractures after the PVP. Ahn Y et al^[36]^ found that low BMI is a risk factor for new hip and spinal fractures. Conversely, vertebral fractures are more likely to occur in overweight patients.^[37]^ The present meta-analysis shows that BMI is not a relevant risk factor. Theoretically, the elderly women are more likely to develop osteoporosis, which could result in vertebral secondary fractures following PVP operation. However, from our meta-analysis, the age and gender are not an independent risk factor. It may be due to the relatively small sample size of the included literatures, the conclusion is still to be proved.

Our research has the following limitations:

1. the included literatures were retrospectively studies.
2. The follow-up time of each study was different and the data collection was incomplete.
3. The quality of the collected documents is uneven.

## 5 Conclusion

Bone mineral density, cement leakage and kyphosis after primary operation are the risk factors closely correlative to the secondary fracture after PVP. There has not been enough evidence to support the associations between the secondary fracture and gender, age, body mass index, cement volume, thoracolumbar spine, and cement injection approaches.

## Acknowledgments

The authors would like to thank Henan People's Hospital of Henan University for providing the database.

## Author contributions

**Data curation:** GongWei Zhai.

**Formal analysis:** Ang Li, Binfeng Liu.

**Funding acquisition:** YanZheng Gao.

**Investigation:** Weichao Sheng.

**Methodology:** Guang Yang.

**Software:** Dongbo Lv, Jingyi Zhang.

**Writing – original draft:** GongWei Zhai.

**Writing – review & editing:** GongWei Zhai, YanZheng Gao.
